# Incidence and outcomes of postoperative paralytic ileus after cytoreductive surgery with HIPEC: A population‐based study

**DOI:** 10.1111/codi.70567

**Published:** 2026-08-04

**Authors:** Lana Ghanipour, Lana Othman Mahmmud, Biying Huang, Gabriella Jansson Palmer, Peter Cashin, Mirna Abraham‐Nordling

**Affiliations:** ^1^ Department of Surgical Sciences Uppsala University Uppsala Sweden; ^2^ Department of Surgery Uppsala University Hospital Uppsala Sweden; ^3^ Department of Pelvic Cancer, Division of Coloproctology, Center for Digestive Diseases Karolinska University Hospital Stockholm Sweden; ^4^ Division of Surgery and Oncology, Department of Clinical Science, Intervention and Technology Karolinska Institute Stockholm Sweden; ^5^ Department of Molecular Medicine and Surgery Karolinska Institutet Stockholm Sweden

**Keywords:** cytoreductive surgery, HIPEC, peritoneal carcinomatosis, postoperative complications, prolonged postoperative ileus

## Abstract

**Background:**

Cytoreductive surgery (CRS) with hyperthermic intraperitoneal chemotherapy (HIPEC) is standard treatment for peritoneal metastases in Sweden. Prolonged postoperative ileus (PPOI) is a common complication following major abdominal surgery and is associated with increased morbidity and prolonged hospitalization. The aim of this study was to evaluate the incidence of PPOI following CRS‐HIPEC and to identify factors associated with its development, including its relationship to postoperative complications.

**Methods:**

This retrospective, bicentre cohort study included patients undergoing CRS‐HIPEC for peritoneal metastases between January 2012 and 2024. Patients were identified through the Swedish HIPEC Registry, with supplementary data obtained from medical records. PPOI was defined as delayed defaecation beyond postoperative day 5 and/or prolonged use or reinsertion of a nasogastric tube. Clinical, surgical and postoperative outcomes were analysed and risk factors for PPOI were assessed using a multivariable logistic regression model.

**Results:**

A total of 982 patients were included, of whom 443 (45%) developed PPOI. Patients with PPOI had significantly longer hospital stays (median 11 vs. 9; *p* < 0.001) and delayed return of bowel function compared with patients without PPOI (median 6 vs. 3 days; *p* < 0.001). Major complications were more frequently observed in patients with PPOI (36% vs. 16%, *p* < 0.001). Independent risk factors for PPOI were major postoperative complications (aOR 3.74, CI 2.55–5.48), blood transfusion (aOR 1.70, CI 1.08–2.70) and omentectomy below the gastroepiploic arcade (aOR 1.51, CI 1.01–2.26). This association remained even after excluding patients with major postoperative surgical complications. Ileostomy formation was associated with a reduced risk of PPOI (aOR 0.51, CI 0.33–0.77).

**Conclusions:**

PPOI is a frequent complication following CRS‐HIPEC and is associated with increased postoperative morbidity and prolonged recovery. Strategies aimed to optimize perioperative care and enhanced recovery protocols may help to reduce the incidence and the adverse effects of PPOI.

**What does this paper add to the literature?**

This population‐based study evaluates PPOI after CRS‐HIPEC in a large cohort, identifies independent predictors and shows that PPOI can arise even without major complications, providing clinically relevant insight into postoperative gastrointestinal recovery.

## INTRODUCTION

Colorectal cancer (CRC) is the third most commonly diagnosed malignancy globally. The peritoneum represents the second most frequent site of metastatic spread from colorectal cancer [1, 2]. Peritoneal metastases (PM) arise in approximately 10% of patients and may either occur synchronously at the time of diagnosis or metachronously during disease progression [3].

For selected patients with peritoneal malignancy, cytoreductive surgery (CRS) combined with hyperthermic intraperitoneal chemotherapy (HIPEC) is an established treatment modality. Despite its therapeutic potential, this extensive surgical procedure is associated with several postoperative complications, among which postoperative paralytic ileus (POI) is common. POI is associated with significant patient discomfort, including absence of flatus, abdominal distension, nausea and vomiting, lack of normal bowel sounds and delayed passage of stool. These complications often result in prolonged hospital stays, increased rates of readmission and occasionally the need for reoperation and higher mortality [4, 5, 6, 7, 8].

Although POI may develop after any abdominal procedure, its incidence is particularly high following major open surgery [9, 10, 11]. In some patients, POI persists beyond the expected recovery period and progresses to prolonged postoperative ileus (PPOI), which is generally defined as a lack of gastrointestinal function for more than 5 days postoperatively [12]. The introduction of minimally invasive surgical techniques has likely reduced the incidence of POI, as laparoscopic procedures are associated with less tissue trauma, decreased inflammatory response and reduced manipulation of the bowel and enteric nervous system [13]. Nevertheless, open surgery remains necessary in a subset of patients, including those with advanced malignancy, recurrent disease or extensive peritoneal metastases.

In this study, we aimed to evaluate the incidence of PPOI lasting beyond postoperative day five following CRS‐HIPEC for peritoneal metastases of various origins. In addition, the study aimed to identify risk factors associated with PPOI, including its association with postoperative complications. A secondary aim was to investigate whether the association between PPOI and outcomes persists in patients undergoing CRS‐HIPEC after adjustment for, or exclusion of, postoperative surgical complications.

## METHOD

### Study design and population

This population‐based cohort study included patients with PM, originating from colorectal cancer, appendiceal adenocarcinoma, pseudomyxoma peritonei (PMP), mesothelioma and small intestine cancer, who underwent CRS‐HIPEC.

**FIGURE 1:**
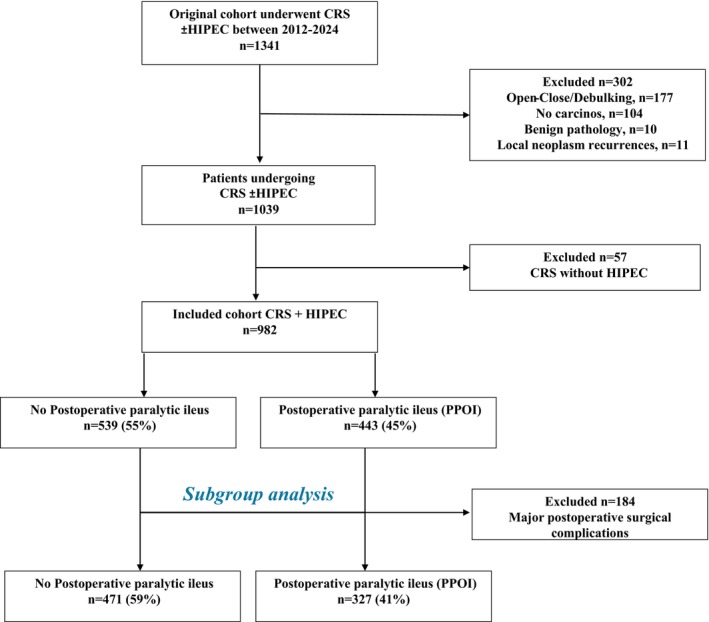
Flowchart of the study.

Patients were identified through the Swedish HIPEC Registry, a nationwide prospective registry with high coverage and data accuracy. Data were collected from two hospital cohorts via the Swedish HIPEC Registry. Patients aged 18 years or older who underwent CRS‐HIPEC between January 2012 and the end of the study period were eligible for inclusion (Figure 1). Follow‐up was censored in December 2025 for patients treated at Uppsala University Hospital and in November 2025 for those treated at Karolinska University Hospital. Information from the registry was complemented by a detailed review of patient medical records, as PPOI is not recorded in the Swedish HIPEC Registry.

Demographic and clinical variables included age, gender, body mass index (BMI), American Society of Anaesthesiologists (ASA) classification, diabetes mellitus and previous abdominal surgery.

Surgical variables included duration of surgery, intraoperative blood loss, peritoneal cancer index (PCI), completeness of cytoreduction (CC score), type of HIPEC regimen (oxaliplatin, irinotecan, mitomycin C or cisplatin), number of organ resections, bowel resections, number and type of anastomoses (entero‐entero, ileo‐colonic, colo‐colonic, colorectal), stoma formation, extent of omentectomy (above or below the gastroepiploic vessels) and number of organ resections.

Postoperative data included duration of nasogastric tube management, time to first defaecation, reinsertion of nasogastric tube, length of hospital stay and in‐hospital complications according to the Clavien‐Dindo classification [14].

A subgroup analysis was conducted to assess the occurrence of PPOI among patients without major postoperative surgical complications (Clavien‐Dindo grade II–IV), in order to explore whether PPOI may occur independently of postoperative surgical complications, such as abdominal reoperations, bile duct leakage, pancreatic leakage and intra‐abdominal abscess or haematoma.

Patients treated at Uppsala University Hospital routinely received Betapred® and prophylactic antibiotic with cefuroxime® for 2 days duration postoperatively, whereas this was not part of standard postoperative management at Karolinska University Hospital.

All patients received thoracic epidural analgesia, and since 2021, a modified enhanced recovery after surgery (ERAS) protocol has been implemented at Uppsala University Hospital [15, 16].

### Exclusion

Patients were excluded if they underwent debulking surgery, open/close surgery or CRS without HIPEC; if intraoperative findings showed no PM or local neoplasm recurrences only; and in cases of benign histopathology.

### CRS and HIPEC procedure

Cytoreductive surgery was performed according to well‐established clinical guidelines [17]. During abdominal exploration, the extent of PM was evaluated using the PCI score and followed by completion of CRS, the completeness of cytoreduction recorded using the CC score [18, 19].

HIPEC was performed using either semi‐open ‘Colosseum method’ or closed abdominal technique. The standard HIPEC regimens used in Sweden include oxaliplatin (460 mg/m^2^), irinotecan (460–500 mg/m^2^), mitomycin C (30 mg/m^2^) or cisplatin (50–100 mg/m^2^). Oxaliplatin and irinotecan were administered for 30 min, while mitomycin C and cisplatin±doxorubicin–based regimens were perfused for 90 and 60 min, respectively.

Intraperitoneal temperature was maintained between 41° and 43°C. A single dose of intravenous 5‐FU (400 mg/m^2^) was given 30 min before the administration of oxaliplatin or irinotecan, followed by folinic acid (60 mg/m^2^). Tiosulfate was given as a bolus dose for nephroprotection at the time of administration of cisplatin ± doxorubicin and continued for 6 h.

### Statistical analysis

Continuous variables are presented as median with interquartile range (IQR), and categorical variables as counts and percentages. Differences between patients with and without PPOI were assessed using the Mann–Whitney *U* test for continuous variables and the chi‐squared test for categorical variables, as appropriate.

Variables considered clinically relevant or showing an association with PPOI in the univariable analyses were entered into a multivariable logistic regression model to identify factors independently associated with PPOI. Adjusted odds ratios (aORs) with 95% confidence intervals (CIs) were calculated. A two‐sided *p*‐value less than 0.05 was considered statistically significant. Statistical analyses were performed using SPSS version 30 (SPSS Inc. Chicago, Illinois, USA).

### Outcomes

The primary outcome was incidence of PPOI, defined as more than 5 days from surgery without defaecation, use of nasogastric tube for more than 5 days or reinsertion of nasogastric tube following surgery.

Secondary outcomes included clinical and surgical factors associated with PPOI, major postoperative complications, CD ≥3a, length of hospital stays (LOS) and time to removal of the nasogastric tube.

## RESULTS

### Patient characteristics

A total of 982 patients underwent CRS‐HIPEC during the study period. Among them, 443 (45%) patients developed PPOI, while 539 (55%) patients did not develop PPOI. Patients with PPOI were older than those without PPOI (median 62 years; *p* = 0.026), whereas gender, BMI, diabetes mellitus and ASA classification were similar between the groups (Table 1).

**TABLE 1:** Clinicopathological characteristics in patients undergoing CRS‐HIPEC.

Patient characteristics	CRS ‐ HIPEC	No PPOI	PPOI	*p*‐value
	*n* = 982 (%)	*n* = 539 (%)	*n* = 443 (%)	
Age (Median)				
≤62 years	515 (52)	200 (37)	215 (49)	**0.026**
≥63 years	467 (48)	239 (44)	228 (51)	
Gender				
Men	441 (45)	236 (44)	205 (46)	0.435
Women	541 (55)	303 (56)	238 (54)	
Body mass index				
Median (IQR)	25 (23–29)	25 (23–28)	26 (23–29)	0.190
Missing	11 (1)	5 (1)	6 (1)	
Diabetes mellitus				
No	898 (91)	497 (92)	401 (91)	0.253
Yes	82 (8)	40 (7)	42 (9)	
Missing	2 (<1)	2 (<1)	0 (0)	
ASA classification				
1	153 (16)	82 (15)	71 (13)	0.519
2	579 (59)	311 (58)	268 (50)	
3	240 (24)	141 (26)	99 (18)	
4	9 (1)	4 (<1)	5 (<1)	
Missing	1 (<1)	1 (<1)	0 (0)	
Previous abdominal surgery				
No	254 (29)	117 (22)	137 (31)	**0.001**
Yes	728 (74)	422 (78)	306 (69)	
Re‐CRS + HIPEC				
No	890 (91)	473 (88)	417 (94)	**<0.001**
Yes	91 (9)	65 (12)	26 (6)	
Missing data	1 (<1)	1 (<1)	0 (0)	
Type of malignancy				
Appendiceal cancer	118 (12)	64 (12)	54 (12)	**0.037**
Colorectal cancer	536 (55)	316 (58)	220 (50)	
Pseudomyxoma peritonei	273 (28)	129 (24)	144 (33)	
Mesothelioma	26 (3)	15 (3)	11 (2)	
Others	29 (3)	15 (3)	14 (3)	
PCI				
Median (Range)	11 (5–19)	11 (6–19)	11 (5–20)	
≤20	765 (78)	431 (80)	334 (75)	0.068
≥21	213 (22)	105 (19)	108 (24)	
Missing data	4 (<1)	3 (<1)	1 (<1)	
CC score				
0	837 (85)	477 (88)	360 (81)	**0.002**
1	135 (14)	55 (10)	80 (18)	
2	7 (<1)	4 (<1)	3 (<1)	
Missing data	3 (<1)	3 (<1)	0 (0)	
Type of HIPEC				
Oxaliplatin	637 (65)	357 (66)	280 (63)	**<0.001**
Irinotecan	162 (16)	114 (21)	48 (11)	
Mitomycin C	153 (16)	56 (10)	97 (22)	
Cisplatin	29 (3)	12 (2)	17 (4)	
Unknown	1 (<1)	0 (0)	1 (<1)	

Abbreviations: CC, completeness of cytoreduction; IQR, interquartile range; PCI, peritoneal cancer index.

Previous abdominal surgery was more common among patients without PPOI (78% vs. 69%, *p* = 0.001), as were repeat CRS‐HIPEC procedures (12% vs. 6%, *p* < 0.001). The distribution of underlying malignancies differed between groups (*p* = 0.037), with PMP being more frequent among patients with PPOI and colorectal PM more common among those without PPOI. Disease burden, measured by PCI, was comparable between groups (median 11 in both; *p* = 0.068). However, CC1 occurred more frequently in patients who developed PPOI (18% vs. 10%, *p* = 0.002), (Table 1).

Differences were also observed in the type of intestinal anastomosis (*p* = 0.041) and the extent of omentectomy (*p* < 0.001). Stoma formation varied significantly between groups, with colostomy being more frequent among patients with PPOI and ileostomy more common among those without PPOI (Table 2).

**TABLE 2:** Prolonged postoperative ileus and surgical outcomes.

	Total (*n* = 982) (%)	No PPOI (*n* = 539) (%)	PPOI (*n* = 443) (%)	*p*‐value
Duration of surgery (ml; IQR)	412 (312–528)	429 (325–540)	390 (300–506)	**<0.001**
Intraoperative blood loss (ml; IQR)	500 (200–1200)	600 (250–1200)	500 (200–1000)	**0.037**
Bowel resection				
No	178 (18)	91 (17)	87 (20)	
Yes	803 (82)	447 (83)	356 (80)	0.271
No. of intestine anastomosis				
0	348 (35)	191 (35)	157 (35)	
1	550 (56)	302 (56)	248 (56)	
2	70 (7)	38 (7)	32 (7)	
3	11 (1)	7 (1)	4 (1)	
4	3 (<1)	1 (<1)	2 (<1)	0.923
Type of anastomosis				
Enteroenteric	142 (14)	77 (14)	65 (15)	
Ileocolic	373 (38)	188 (35)	185 (42)	
Colocolic	23 (2)	12 (2)	11 (2)	
Colorectal	153 (16)	100 (19)	53 (12)	
Ileosigmoid or ileorectal	16 (2)	8 (1)	8 (2)	**0.041**
Type of stoma				
No stoma	499 (51)	243 (45)	256 (58)	
Ileostomy	300 (31)	210 (39)	90 (20)	
Colostomy	182 (19)	85 (16)	97 (22)	
Missing data	1 (<1)	1 (<1)	0 (0)	**<0.001**
Omentectomy				
Above a gastroepiploic	610 (62)	356 (66)	254 (57)	
Below a gastroepiploic	222 (23)	92 (17)	130 (29)	
Previously resected	104 (11)	59 (11)	45 (10)	
Not resected	41 (4)	30 (6)	11 (2)	
Unknown	5 (<1)	2 (<1)	3 (<1)	**<0.001**
No of organ resections				
1–3	241 (24)	129 (24)	112 (25)	
4–6	478 (49)	271 (50)	207 (47)	
≥7	263 (27)	139 (26)	124 (28)	0.535
Days to first defecation, median (IQR)		3 (2–4)	6 (5–9)	**<0.001**
Days with nasogastric tube (IQR)		1 (1–2)	2 (1–4)	**<0.001**
New nasogastric tube placed, *n* (%)		NA	153 (35)	
Duration of new nasogastric tube (days), median (IQR)^a^		NA	3 (2–6)	
Missing data, *n*		0	2	
Length of hospital stay (days)				
Median (IQR)	10 (8–14)	9 (7–12)	11 (8–16)	
Missing data	5 (<1)	5 (<1)	0 (0)	**<0.001**

Abbreviation: NA, not applicable.

^a^ Duration calculated from tube insertion to removal or transfer to referring hospital. Patients with gastrostomy were excluded (*n* = 12).

### Postoperative outcomes

Patients who developed PPOI had a longer hospital stay (median 11 days [IQR 8–16 days]), compared with patients without PPOI (median 9 days [IQR 7–12]; *p* < 0.001) (Table 2).

**FIGURE 2:**
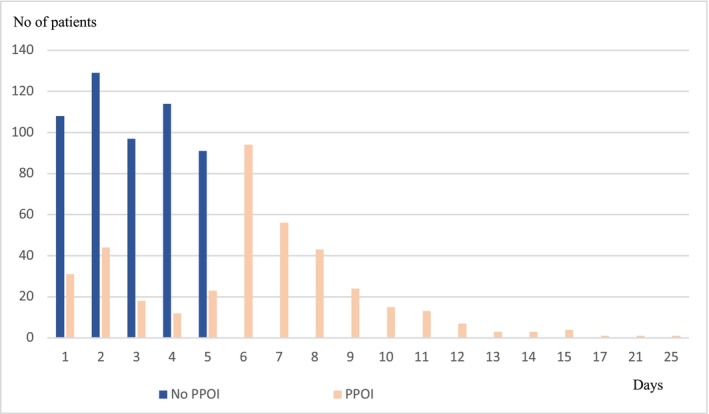
Number of patients according to day of first postoperative defaecation in patients with or without prolonged postoperative ileus.

Return of bowel function was significantly delayed in patients with PPOI (Table 2). The median time to first defaecation was 6 days (IQR 5–9) compared with 3 days (IQR 2–4) among patients without PPOI (*p* < 0.001). The distribution of patients according to postoperative day of first defaecation is shown in Figure 2.

The use of nasogastric tube was also prolonged in the PPOI group (median 2 days [IQR 1–4] vs. 1 day [IQR 1–2]; *p* < 0.001). In addition, 153 patients (35%) with PPOI required placement of a new nasogastric tube. Among these, the median duration of the newly inserted tube was 3 days (IQR 2–6). Patients with gastrostomy (*n* = 12) were excluded from this analysis (Table 2).

### Postoperative complications

Postoperative morbidity was significantly higher among patients with PPOI. Major complications (CD grade III–IV) occurred in 36% of patients with PPOI compared with 16% among patients without PPOI (*p* < 0.001). Mortality was low and similar between the groups (0.5% vs. 0.7%). Reoperation was more frequent in the PPOI group (17% vs. 5%, *p* < 0.001) (Table 3).

**TABLE 3:** Postoperative complications.

Complication type	No PPOI (*n* = 539) (%)	PPOI (*n* = 443) (%)	*p*‐value
Clavien‐Dindo Grade			
I–II	451 (83.7)	280 (63.2)	**<0.001**
III–IV	84 (15.6)	161 (36.3)	
V	4 (0.7)	2 (0.5)	
Reoperation			
Yes	25 (4.6)	75 (16.9)	**<0.001**
No	514 (95.4)	366 (82.3)	
Missing data	0 (0.0)	2 (0.5)	
Any surgical complications	79 (15)	189 (42.7)	**<0.001**
Intra‐abdominal abscess	33 (6.1)	40 (9.0)	0.084
Bleeding/haematoma	13 (2.4)	18 (4.1)	0.141
Gastrointestinal perforation	3 (0.6)	22 (5.0)	**<0.001**
Urinary tract injury	4 (0.7)	5 (1.1)	0.527
Anastomotic leakage	4 (0.7)	17 (3.8)	**0.001**
Wound dehiscence	9 (1.7)	22 (5.0)	**0.003**
Rectal stump blow‐out	0 (0.0)	4 (0.9)	**0.027**
Pneumothorax	2 (0.4)	11 (2.5)	**0.004**
Enterocutaneous fistula	1 (0.2)	1 (0.2)	0.889
Biliary leakage	4 (0.7)	7 (1.6)	0.214
Others	21 (3.9)	24 (5.4)	0.257
Any medical complications	148 (27)	169 (38)	**<0.001**
Pneumonia	11 (2.0)	16 (3.6)	0.134
Pleural effusion	27 (5.0)	53 (12.0)	**<0.001**
Thromboembolism	15 (2.8)	22 (5.0)	0.073
Cardiac arrhythmia	13 (2.4)	16 (3.6)	0.269
Kidney failure	6 (1.1)	13 (2.9)	**0.039**
Neutropenia	34 (6.3)	36 (8.1)	0.273
Sepsis	10 (1.9)	24 (5.4)	**0.002**
Unknown infection	32 (5.9)	31 (7.0)	0.499
Urinary tract infection	18 (3.3)	11 (2)0.5	0.430
Transfusion	64 (11.8)	99 (22.3)	**<0.001**

Overall, surgical complications occurred in 43% of patients with PPOI compared with 15% among those without PPOI (*p* < 0.001). Gastrointestinal perforation (5% vs. 0.6%), anastomotic leakage (3.8% vs. 0.7%) and wound dehiscence (5% vs. 1.7%) were more common among patients with PPOI. Medical complications were also more frequent in the PPOI group (38% vs. 27%, *p* < 0.001). Pleural effusion (12% vs. 5%), kidney failure (3% vs. 1%), sepsis (5% vs. 2%) and postoperative blood transfusion (22% vs. 12%) occurred more often in patients with PPOI (Table 3).

### Factors associated with prolonged postoperative ileus

Major postoperative complications (CD grade III–IV) were strongly associated with the development of PPOI (aOR 3.74, CI 2.55–5.48), in a multivariable logistic regression model adjusted for age, repeat CRS‐HIPEC procedures, PCI, CC score, intraoperative blood loss and HIPEC regimen. Blood transfusion (aOR 1.70, CI 1.08–2.70) and omentectomy below the gastroepiploic arcade (aOR 1.51, CI 1.01–2.26) were also independently associated with PPOI (Table S1).

In contrast, ileostomy formation was associated with a lower risk of PPOI (aOR 0.51, CI 0.33–0.77). Similarly, duration of surgery ≥413 min was inversely associated with PPOI (aOR 0.64, CI 0.42–0.97).

### PPOI in patients without major postoperative complications

A subgroup analysis including patients (*n* = 798) without major postoperative surgical complications (Clavien‐Dindo grade II–IV), in order to explore whether prolonged PPOI may occur independently of postoperative morbidity. In this subgroup, 327 (41%) had PPOI.

Factors independently associated with an increased risk of PPOI were omentectomy below the gastroepiploic arcade (aOR 1.62, 95% CI 1.06–2.48; *p* = 0.025), Mitomycin C (aOR 1.83, 95% CI 1.03–3.24; *p* = 0.039) and postoperative blood transfusion (aOR 1.93, 95% CI 1.16–3.22; *p* = 0.012) (Table 4).

**TABLE 4:** Uni‐ and multivariable logistic regression analyses of factors associated with prolonged postoperative ileus. Major postoperative surgical complications were excluded (*n* = 184).

	Total *n* = 798	Univariate OR (95% CI)	*p*‐value	Adjusted OR^a^ (95% CI)	*p*‐value
Age					
≤62 years	471	Ref.			
≥63 years	327	1.30 (0.98–1.72)	0.071		
Diabetes mellitus					
No	735	Ref.			
Yes	61	1.07 (0.63–1.82)	0.799		
Primary tumour					
Appendiceal cancer	88	Ref.		Ref.	
Colorectal cancer	437	0.92 (0.57–1.47)	0.717	1.17 (0.65–2.11)	0.601
Pseudomyxoma peritonei	225	1.81 (1.09–2.99)	**0.022**	1.60 (0.84–3.07)	0.156
Mesothelioma	24	1.41 (0.57–3.51)	0.460	0.79 (0.16–3.87)	0.786
Others	24	1.41 (0.57–3.51)	0.460	2.55 (0.85–7.68)	0.095
Re‐CRS + HIPEC					
No	471	Ref.		Ref.	
Yes	327	0.58 (0.34–0.98)	**0.042**	6.80 (0.79–58.63)	0.081
Omentectomy					
Above gastroepiploic	478	Ref.		Ref.	
Below gastroepiploic	200	2.30 (1.65–3.22)	**<0.001**	1.62 (1.06–2.48)	**0.025**
PCI					
≤20	630	Ref.			
≥21	164	1.27 (0.90–1.80)	0.173		
Bowel resections					
Yes	158	Ref.		Ref.	
No	639	0.68 (0.48–0.96)	**0.028**	0.74 (0.43–1.25)	0.257
Number of resections					
1–3	215	Ref.			
4–6	391	0.77 (0.55–1.08)	0.123		
≥7	192	0.78 (0.53–1.16)	0.222		
Type of stoma					
None	422	Ref.		Ref.	
Ileostomy	233	0.33 (0.23–0.48)	**<0.001**	0.47 (0.28–0.77)	**0.003**
Colostomy	143	1.12 (0.76–1.63)	0.529	1.26 (0.75–2.09)	0.383
HIPEC regimen					
Oxaliplatin	502	Ref.		Ref.	
Irinotecan	127	0.63 (0.42–0.96)	**0.029**	0.66 (0.38–1.14)	0.136
Mitomycin C	132	2.78 (1.87–4.14)	**<0.001**	1.83 (1.03–3.24)	**0.039**
Cisplatin	26	2.24 (1.01–4.98)	**0.048**	2.61 (0.70–9.69)	0.153
Duration of surgery (min)					
≤412 min	402	Ref.		Ref.	
≥413 min	351	0.53 (0.39–0.71)	**<0.001**	0.71 (0.44–1.12)	0.142
Blood loss (ml)					
≤500 mL	406	Ref.		Ref.	
≥501 mL	347	0.69 (0.52–0.93)	**0.014**	1.21 (0.78–1.85)	0.397
Transfusion					
No	688	Ref.		Ref.	
Yes	110	1.98 (1.32–2.96)	**0.001**	1.93 (1.16–3.22)	**0.012**

^a^ Adjusted for primary tumour, repeat CRS‐HIPEC procedures, omentectomy, bowel resections, type of stoma formation, HIPEC regimen, duration of surgery, intraoperative blood loss and transfusion.

## DISCUSSION

In this large population‐based cohort of patients undergoing CRS‐HIPEC, PPOI occurred in nearly half of all patients (45%), underscoring that impaired gastrointestinal recovery remains a major postoperative challenge in this setting. PPOI was associated with a substantial increase in postoperative morbidity, including prolonged hospital stay. Major postoperative complications emerged as the strongest independent factor associated with PPOI, suggesting a close and potentially bidirectional relationship between postoperative complications and impaired gastrointestinal recovery. Conversely, construction of an ileostomy appeared to reduce the likelihood of PPOI. When patients with postoperative surgical complications were excluded, the incidence of PPOI decreased to 41%, indicating that although complications contribute significantly to PPOI, a considerable proportion of cases occur independently of overt surgical morbidity.

In a prospective study by Nors et al. investigating the incidence of PPOI after CRS‐HIPEC, a frequency of 54% was reported, with a median time to first defaecation of 6 days postoperatively [20]. The high incidence of PPOI following CRS‐HIPEC is likely multifactorial. The procedure involves extensive peritoneal stripping, multivisceral resections, prolonged operative duration, changes in electrolyte and fluid balance and intraoperative blood loss, all of which contribute to surgical trauma and activation of inflammatory pathways known to impair gastrointestinal motility [21, 22]. In addition, bowel manipulation and exposure to hyperthermic intraperitoneal chemotherapy may further exacerbate intestinal dysmotility, potentially through bowel wall oedema and increased intestinal wall thickness [23].

Millan et al. reported an increased risk of PPOI among patients undergoing colorectal cancer surgery with an ileostomy formation [24]. In contrast, our study demonstrated that ileostomy was associated with a reduced risk of PPOI. This discrepancy may reflect differences in study populations and surgical context, including the greater extent of multivisceral resections and bowel manipulation in CRS‐HIPEC, as well as underlying pathophysiological mechanisms. However, this finding should be interpreted with caution, as stoma formation is influenced by intraoperative findings and surgical strategy.

Evers et al. investigated whether preservation of the right gastroepiploic artery during omentectomy has a positive effect on gastric emptying after CRS‐HIPEC in a randomized study including 42 patients and found no difference between the groups [25]. In our cohort, omentectomy below the gastroepiploic arcade was independently associated with PPOI. Although omentectomy is not directly associated with gastroparesis, more extensive peritoneal and omental resection may reflect increased surgical trauma and inflammatory activation [21, 25, 26].

The protective association of ileostomy observed in our cohort may be related to differences in gastrointestinal motility recovery. Following surgery, small bowel motility typically resumes earlier, whereas colonic motility may be delayed for several days due to increased susceptibility to surgical stress, inflammatory responses and neurogenic inhibition [27]. Furthermore, a diverting stoma may mitigate the clinical consequences of subclinical complications such as low‐grade anastomotic leakage or localized inflammation, both known to impair gastrointestinal motility [27, 28].

The strong association between PPOI and major postoperative complications likely reflects a bidirectional relationship. Complications such as intra‐abdominal abscess, anastomotic leakage or sepsis may impair intestinal dysmotility through inflammatory and metabolic disturbances [29]. Conversely, prolonged ileus may worsen patient outcomes by delaying enteral nutrition, promoting malnutrition, causing electrolyte imbalances and increasing the risk of aspiration [26]. These findings emphasize the importance of strategies aimed at reducing both PPOI and postoperative complications.

A key challenge when comparing studies is the lack of a standardized definition of postoperative ileus (POI). Various criteria have been used, including time to passage of flatus or stool, tolerance of oral intake or need for nasogastric decompression [12]. Quiroga‐Centeno et al. suggested that the most commonly applied cut‐off is ≥4 days after index surgery [29]. However, a meta‐analysis of patients undergoing colorectal surgery reported that the threshold for defining POI varies widely, ranging from 3 to 7 days [30]. In some studies, POI has even been defined solely as the need for reinsertion of a nasogastric tube in patients who were perioperatively decompressed [30, 31].

In our study, PPOI was defined using parameters, including delayed defaecation (>5 days) and prolonged use or reinsertion of nasogastric tube. While this definition likely captures clinically meaningful postoperative gastrointestinal dysfunction, it may limit direct comparability with other cohorts [6, 12, 32, 33].

Enhanced recovery after surgery (ERAS) programmes have been shown to reduce morbidity and length of hospital stay in colorectal and ovarian cancer surgery; however, their impact on postoperative ileus remains inconsistent [6, 33, 34]. In the context of CRS‐HIPEC, a limited number of studies have evaluated ERAS protocols, suggesting improvements in postoperative recovery, including earlier return of gastrointestinal function, reduced postoperative complication rates and shorter hospital stay [35, 36, 37]. However, the available evidence is based primarily on retrospective observational studies.

An ERAS protocol was not uniformly implemented in the present cohort, although a modified version has been introduced at one of the participating centres since 2021. Prospective studies are warranted to determine whether standardized ERAS protocols can reduce the incidence of PPOI in patients undergoing CRS‐HIPEC.

## LIMITATIONS

One limitation of this study is its retrospective observational design, which may introduce inherent bias. Other limitations include differences in patient care between the two centres, including variations in the likelihood of receiving a stoma during CRS‐HIPEC. Furthermore, patients with ileostomies experienced a shorter time to defaecation following surgery. To account for these differences, the authors incorporated both the use and reinsertion of nasogastric tube into the definition of PPOI.

## STRENGTH

One of the main strengths of this study is the population‐based study cohort with a relatively large sample size and a high data completeness, with minimal missing values. Furthermore, all medical records were manually reviewed by experienced colorectal and HIPEC surgeons (the authors) to ensure data accuracy and to identify cases of PPOI, since this variable was not directly captured in the national HIPEC registry.

Another strength is that both centres (Uppsala University Hospital and Karolinska University Hospital) are national referral centres for peritoneal surface malignancies and adhere to standardized national protocols.

## CONCLUSION

Prolonged postoperative ileus is a common complication following CRS‐HIPEC, affecting nearly half of the patients in this cohort. Its strong association with major postoperative complications and prolonged hospital stay highlights its significance as a determinant of postoperative recovery. While ileostomy formation appeared to confer a protective effect, the underlying mechanism remains unclear and warrants further investigation. Future research should also focus on standardizing definitions of PPOI, identifying risk factors, evaluating targeted perioperative strategies, including ERAS protocols, to reduce the incidence and clinical impact of PPOI in patients undergoing CRS‐HIPEC.

## AUTHOR CONTRIBUTIONS

**Biying Huang:** Conceptualization; investigation; validation; data curation; writing – review and editing. **Peter Cashin:** Conceptualization; investigation; validation; writing – review and editing. **Gabriella Jansson Palmer:** Conceptualization; investigation; validation; writing – review and editing. **Lana Ghanipour:** Conceptualization; investigation; funding acquisition; writing – original draft; methodology; writing – review and editing; formal analysis; project administration; data curation; supervision; validation; software. **Lana Othman Mahmmud:** Data curation; investigation; validation; writing – review and editing; conceptualization. **Mirna Abraham‐Nordling:** Conceptualization; investigation; writing – original draft; writing – review and editing; validation; methodology; data curation.

## FUNDING INFORMATION

Provided by grants from Uppsala University Hospital, Uppsala, Sweden.

## CONFLICT OF INTEREST STATEMENT

The authors declare no conflict of interest.

## ETHICS STATEMENT

The study was approved by the local ethics committee of Stockholm (2025–02600‐02).

## GENERATIVE ARTIFICIAL INTELLIGENCE (AI)

ChatGPT (version GPT‐4.1‐mini) was used to assist with language refinements.

## Supporting information


Table S1.

## Data Availability

The dataset used in this study is available on request to the corresponding author.
