# Supplementary material for: Incidence and outcomes of postoperative paralytic ileus after cytoreductive surgery with HIPEC: A population‐based study
Source: Colorectal Dis. 2026 Aug 4;28(8):e70567. doi: 10.1111/codi.70567 (PMC13438104; doi:10.1111/codi.70567)
Supplement: Supplementary file 1 [file CODI-28-0-s001.docx]

Supplementary Table 1. Uni- and multivariable logistic regression analysis of factors associated with prolonged postoperative ileus.

|  | Univariate OR (95% CI) | p-value | Adjusted OR* (95% CI) | p-value |
| --- | --- | --- | --- | --- |
| **Age**  **≤** 62 years  ≥ 63 years | Ref.  1.33 (1.03-1.71) | **0.026** | Ref.  1.31 (0.95-1.82) | 0.100 |
| **Diabetes Mellitus**  No  Yes | Ref.  1.30 (0.83-2.05) | 0.254 |  |  |
| **Primary tumor**  Appendiceal cancer  Colorectal cancer  Pseudomyxoma peritonei  Mesothelioma  Others | Ref.  0.83 (0.55-1.23)  1.32 (0.86-2.04)  0.87 (0.37-2.01)  1.11 (0.49-2.50) | 0.347  0.205  0.749  0.808 |  |  |
| **Re CRS+HIPEC**  No  Yes | Ref.  0.45 (0.28-0.73) | **0.001** | Ref.  2.09 (0.49-8.91) | 0.318 |
| **Omentectomy**  Above gastroepiploic  Below gastroepiploic | Ref.  1.98 (1.45-2.71) | **<0.001** | Ref.  1.51 (1.01-2.26) | **0.044** |
| **PCI**  ≤20  ≥21 | Ref.  1.33 (0.98-1.80) | 0.068 | Ref.  1.32 (0.82-2.12) | 0.258 |
| **Bowel resections**  Yes  No | Ref.  0.83 (0.60-1.15) | 0.271 |  |  |
| **Number of resections**  1-3  4-6  ≥ 7 | Ref.  0.80 (0.52-1.25)  0.86 (0.57-1.29) | 0.327  0.455 |  |  |
| **Type of stoma**  None  Ileostomy  Colostomy | Ref.  0.41 (0.30-0.55)  1.08 (0.77-1.52) | **<0.001**  0.645 | Ref.  0.51 (0.33-0.77)  0.90 (0.57-1.42) | **0.001**  0.636 |
| **HIPEC regimen**  Oxaliplatin  Irinotecan  Mitomycin C  Cisplatin | Ref.  0.54 (0.37-0.78)  2.21 (1.53-3.18)  1.81 (0.85-3.84) | **0.001**  **<0.001**  0.125 | Ref.  0.75 (0.45-1.25)  1.60 (0.99-2.57)  2.01 (0.76-5.35) | 0.267  0.055  0.161 |
| **CC score**  0  1 | Ref.  1.93 (1.33-2.79) | **<0.001** | Ref.  1.55 (0.91-2.66) | 0.109 |
| **Duration of surgery (minutes)**  ≤412 min  ≥413 min | Ref.  0.60 (0.46-0.78) | **<0.001** | Ref.  0.64 (0.42-0.97) | **0.036** |
| **Blood loss (ml)**  ≤ 500 ml  ≥501 ml | Ref.  0.79 (0.61-1.03) | 0.080 | Ref.  1.12 (0.76-1.66) | 0.572 |
| **Transfusion**  No  Yes | Ref.  2.14 (1.52-3.01) | **<0.001** | Ref.  1.70 (1.08-2.70) | **0.023** |
| **Postoperative complication (CD)**  I-II  III-IV | Ref.  3.10 (2.28-4.18) | **<0.001** | Ref.  3.74 (2.55-5.48) | **<0.001** |

* Adjusted for age, repeat CRS-HIPEC procedures, Omentectomy, PCI, HIPEC-regimen, type of stoma formation, CC score, duration of surgery, intraoperative blood loss, transfusion, and postoperative complications.
